# Mitochondrial Dysfunction and Oxidative Stress Promote Apoptotic Cell Death in the Striatum via Cytochrome c/Caspase-3 Signaling Cascade Following Chronic Rotenone Intoxication in Rats

**DOI:** 10.3390/ijms13078722

**Published:** 2012-07-13

**Authors:** Tsu-Kung Lin, Ching-Hsiao Cheng, Shang-Der Chen, Chia-Wei Liou, Chi-Ren Huang, Yao-Chung Chuang

**Affiliations:** 1 Department of Neurology, Kaohsiung Chang Gung Memorial Hospital and Chang Gung University College of Medicine, Kaohsiung 833, Taiwan; E-Mails: chensd@adm.cgmh.org.tw (S.-D.C.); cwliou@ms22.hinet.net (C.-W.L.); kcn68@ms22.hinet.net (C.-R.H.); 2 Center for Parkinson’s Disease, Kaohsiung Chang Gung Memorial Hospital and Chang Gung University College of Medicine, Kaohsiung 833, Taiwan; 3 Neurosurgery, Kaohsiung Chang Gung Memorial Hospital and Chang Gung University College of Medicine, Kaohsiung 833, Taiwan; E-Mail: ma4200@adm.cgmh.org.tw; 4 Center for Translational Research in Biomedical Sciences, Kaohsiung Chang Gung Memorial Hospital and Chang Gung University College of Medicine, Kaohsiung 833, Taiwan; 5 Department of Biological Science, National Sun Yet-Sen University, Kaohsiung 804, Taiwan

**Keywords:** rotenone, Parkinson’s disease, mitochondria, complex I, apoptotic cell death, striatum

## Abstract

Parkinson’s disease (PD) is a progressive neurological disorder marked by nigrostriatal dopaminergic degeneration. Evidence suggests that mitochondrial dysfunction may be linked to PD through a variety of different pathways, including free-radical generation and dysfunction of the mitochondrial Complex I activity. In Lewis rats, chronic systemic administration of a specific mitochondrial Complex I inhibitor, rotenone (3 mg/kg/day) produced parkinsonism-like symptoms. Increased oxidized proteins and peroxynitrite, and mitochondrial or cytosol translocation of Bim, Bax or cytochrome c in the striatum was observed after 2–4 weeks of rotenone infusion. After 28 days of systemic rotenone exposure, imunohistochemical staining for tyrosine hydroxylase indicated nigrostriatal dopaminergic neuronal cell degeneration. Characteristic histochemical (TUNEL or activated caspase-3 staining) or ultrastructural (electron microscopy) features of apoptotic cell death were present in the striatal neuronal cell after chronic rotenone intoxication. We conclude that chronic rotenone intoxication may enhance oxidative and nitrosative stress that induces mitochondrial dysfunction and ultrastructural damage, resulting in translocation of Bim and Bax from cytosol to mitochondria that contributes to apoptotic cell death in the striatum via cytochrome c/caspase-3 signaling cascade.

## 1. Introduction

Parkinson’s disease (PD) is the second most common neurodegenerative disease in the world, affecting about 1% of adults older than 60 years [1]. PD is a chronic, progressive disease caused by degeneration of specific neuronal population in the brain, notably the dopaminergic neurons of the substantia nigra pas compacta [1,2]. With the increasing age of the general population, the prevalence of PD will rise steadily in the future [1]. The impact of this disease indicates that PD patients have a two to five-fold higher risk of mortality than the general population [3,4].

Increasing evidence suggests that mitochondrial dysfunction may be linked to PD through a variety of pathways, including free-radical generation, inflammation, deficiency of the activity of mitochondrial respiratory chain enzyme Complex I [5–7]. Evidence showed that mitochondrial Complex I dysfunction and oxidative stress play a crucial role in the pathogenesis of PD [5–9]. This is also supported by the fact that in patients with PD have a 30% to 40% decrease in mitochondrial Complex I activity in the substantia nigra pars compacta [10,11]. This dysfunction of mitochondrial respiratory chain can eventually lead to both apoptotic and necrotic neuronal cell death [12–14].

Activation of mitochondrion-dependent apoptotic cell death pathways is instrumental to the demise of substantia nigra pars compacta dopaminergic neurons in experimental mouse models of PD [12–14]. Mitochondrial Complex I toxins, such as rotenone can induce dopaminergic cell death and produce a parkinsonian state in experimental animals [6,15–17]. Rotenone is a commonly used, naturally occurring, organic pesticide, and also a classical, high affinity inhibitor of mitochondrial Complex I [6,15,16]. Again, with extreme lipophilicity, it crosses biological membranes easily and gets into the brain rapidly. As such, it is well-suited for inducing a systemic inhibition of Complex I in experimental animal model of PD [17–20]. It has been shown that chronic systemic Complex I inhibition caused by rotenone exposure induces parkinsonism features in rats, including selective nigrostriatal dopaminergic degeneration and formation of ubiquitin- and alpha-synuclein-positive inclusions [21]. Besides degeneration of substantia nigra pars compacta, evidence also showed degeneration in other brain areas under chronic systemic rotenone exposure, such as the striatum and prefrontal cortex [18,22]. However, there is still a lack of understanding of the exact mechanism of striatal dopamine neuronal cell death in the animal model of PD induced by rotenone. Therefore, the present study carried on the rat model that under chronic systemic exposure of rotenone which has been proved to be able to induce a parkinsonian state [17,23,24] for recapitulate the mechanism of mitochondrial related cell death pathway in the striatum under chronic systemic rotenone intoxication.

## 2. Results and Discussion

### 2.1. Behavior, Body Weight and Mortality Following Chronic Rotenone Intoxication

Behaviorally, 47% of rotenone-infused rats exhibited Parkinsonism-like symptoms that included reduced mobility, flexed posture and rigidity. We routinely selected the symptomatic rats in this study; otherwise, the asymptomatic animals were not included. Compared with sham controls, significantly reduced body weight gain was observed in animals with chronic rotenone intoxication. The body weight gain was 13.2% less than sham control rats on day 7, 12.5% on day 14, 10.9% on day 21 and 9.6% on day 28. The mortality rate in the rotenone treated rats was 25%.

### 2.2. Increase of Oxidative and Nitrosative Stress in the Striatum under the Chronic Rotenone Intoxication

**Figure 1:**
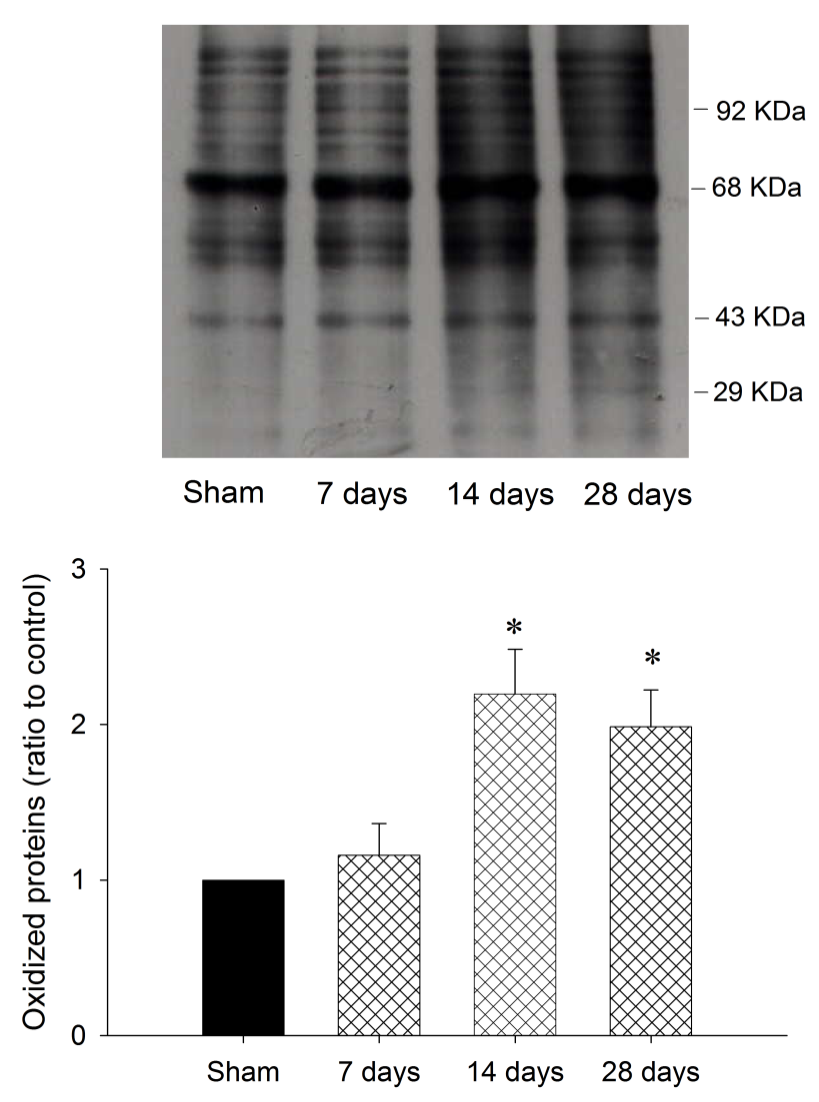
Representative gels (inset) or temporal changes of protein oxidation detected in samples collected from the striatum 7, 14, and 28 days after systemic infusion of rotenone (3 mg/kg/day). Total proteins were extracted from striatum at indicted times or from sham-operated controls followed by immunoblot analysis for the extent of protein oxidation. Values in the lower panel are fold changes with reference to sham-control (S) and are mean ± SEM of four animals per experimental group. * *p* < 0.05 *versus* sham-control group in the Scheffé multiple-range test.

**Figure 2:**
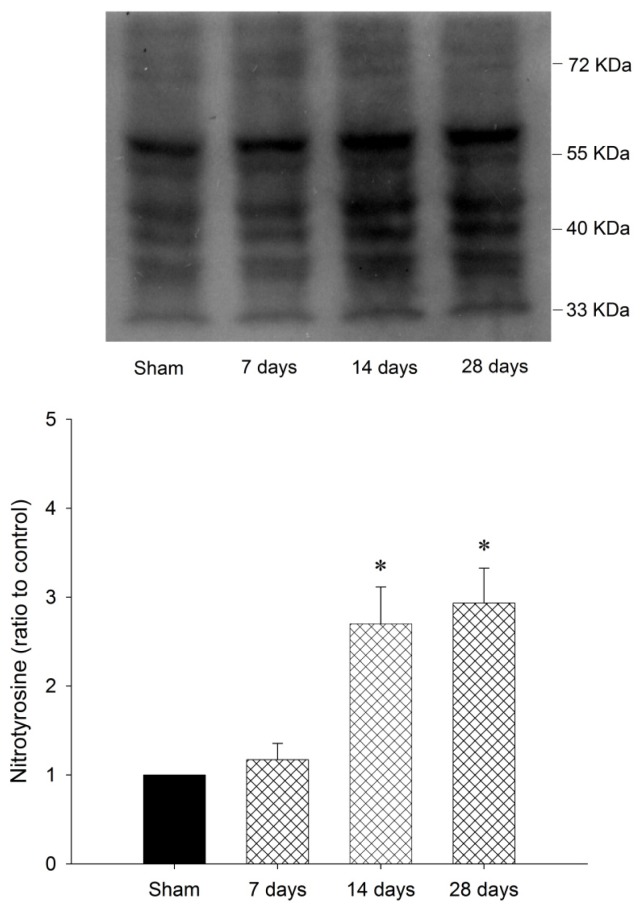
Representative temporal changes in nitrotyrosine (NT) relative to β-actin protein, detected in samples collected from the striatum 7, 14, and 28 days after systemic infusion of rotenone (3 mg/kg/day). Values are mean ± SEM of quadruplicate analyses from 4 to 6 animals per experimental group. * *p* < 0.05 *versus* sham-control group in the Scheffé multiple-range test.

Our first series of experiments established that oxidative and nitrosative stress damage occurred in the striatal cells under the chronic rotenone intoxication. We observed a significantly heightened content of oxidized proteins (Figure 1) and nitrotyrosine (Figure 2) in the samples obtained from striatum 14 to 28 days under systemic rotenone (3 mg/kg/day) infusion.

### 2.3. Temporal Course of Bim, Bax, Bid and Cytochrome c Translocation in the Striatum during Chronic Rotenone Intoxication

**Figure 3:**
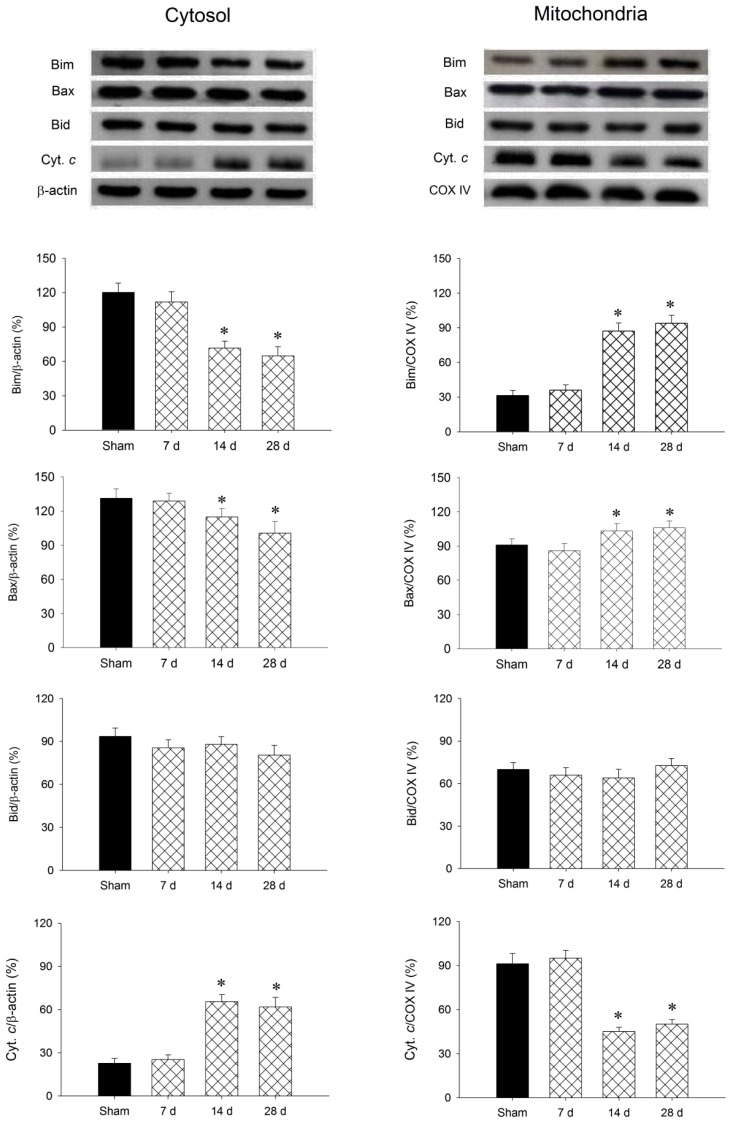
Representative gels (inset) or temporal changes in Bim, Bax, Bid or cytochrome *c* relative to β-actin protein detected in the cytosolic or relative to cytochrome c oxidase subunit IV (COX-IV) in the mitochondrial fraction of samples collected from the striatum 7, 14, and 28 days after systemic infusion of rotenone (3 mg/day/kg). Values are mean ± SEM of quadruplicate analyses from six animals per experimental group. * *p* < 0.05 *versus* sham-control group in the Scheffé multiple-range test.

Our second series of experiments investigated whether the Bim, Bax, Bid and cytochrome c signaling cascades are associated with chronic mitochondrial Complex I inhibition in the striatum following rotenone treatment. Western blot analysis revealed that Bid was not discernible as having altered in either the mitochondrial or cytosolic fraction of samples obtained from the striatum. However, there was a significant decrease of Bim and Bax level and increase of cytochrome c level in the cytosolic fraction (Figure 3A) of samples from the striatum after 2–4 weeks of systemic infusion of rotenone (3 mg/kg/day), accompanied by a corresponding increase of Bim and Bax level and decrease of cytochrome c level in the mitochondrial fraction (Figure 3B).

### 2.4. Neurons with Ultrastructural Features of Apoptosis and Mitochondria Damage in the Striatum Following Chronic Rotenone Intoxication

**Figure 4:**
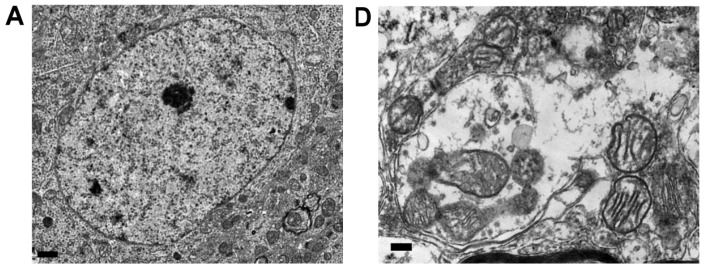

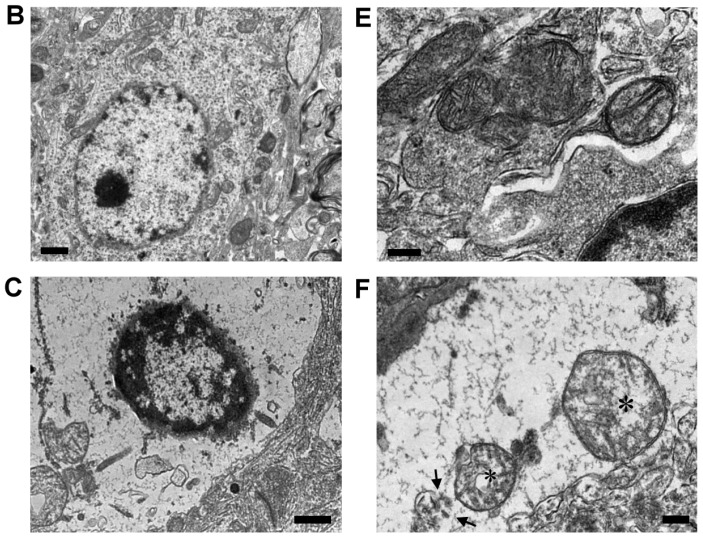
Representative electron photomicrographs of mitochondrial ultrastructure in striatum. (**A**) A pyramidal neuron with intact ultrastructural features in sham-control group; Ultrastructural features of early (**B**) or severe (**C**) apoptotic cell death 14 or 28 days after systemic infusion of rotenone (3 mg/kg/day); (**D**) and (**E**) Higher magnification showed normal mitochondria in in sham-control group and early apoptotic neurons; (**F**) Severe mitochondrial damage was noted 28 days after systemic infusion of rotenone (3 mg/kg/day). Swelling of all mitochondrial spaces, particular in cristae (asterisk). (**F**) Note severe mitochondrial swelling accompanied by a disruption in membrane integrity (arrows). Scale bar: 1 μm in (**A**), (**B**) and (**C**) and 0.5 μm in (**D**), (**E**) and (**F**).

Our third series of experiments determined whether the integrity of the mitochondria was damaged in the striatal neurons that exhibited the ultrastructural features of apoptosis under rotenone intoxication. As exemplified by a neuron in the striatum (Figure 4A), electron microscopy showed oval nuclear morphology, prominent nucleolus, normal cytoplasmic density and normal cytoplasmic organelles, particularly intact utrastracture of mitochondria (Figure 4D) in the striatal neurons in sham control rats. On the other hand, neurons with ultrastructural features of apoptotic cell death were identified in the striatum under systemic rotenone intoxication. After 14 days of rotenone intoxication (3 mg/kg/day), the striatal neurons manifested an early stage of apoptotic changes (Figure 4B). The nucleus was reduced in size but surrounded by an intact membrane. The chromatin was heterochromatic in appearance, with mild margination. Intriguingly, relatively intact mitochondria (Figure 4E) were clearly recognizable in the cytoplasm of striatal neurons that exhibited early apoptotic cell death feature. After 28 days of systemic rotenone intoxication, striatal neurons presented with advanced degree of apoptotic features (Figure 4C) that exhibited a shrinkage appearance of its markedly reduced cell body, alongside increased cytoplasmic electron density and high condensation and margination of chromatin in the much-diminished nucleus. Mitochondrial ultrastructural damage was noted after 28 days of systemic rotenone intoxication that was associated with significant swelling of all mitochondrial spaces, including cristae, and in the advanced cases, mitochondrial swelling was accompanied by a disruption in membrane integrity (Figure 4F).

### 2.5. Dopaminergic Neuronal Cell Loss and Apoptotic Cell Death in the Striatum and Substantia Nigra Following Chronic Rotenone Intoxication

**Figure 5:**
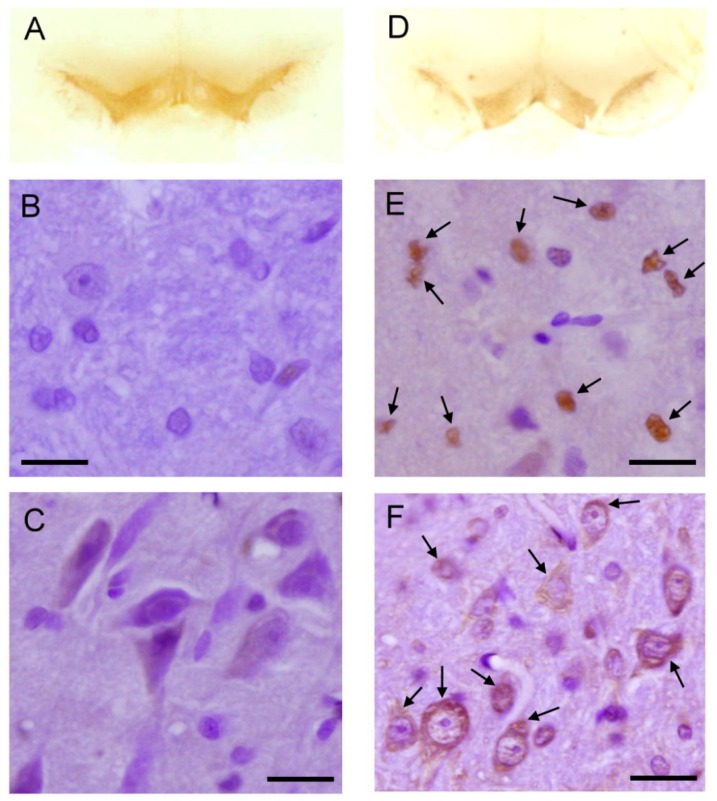
Tyrosine hydroxylase (**A**, **D**), TUNEL (**B**, **E**), and caspase-3 (**C**, **F**) staining of the substantia nigra in sham controls or 28 days of rotenone (3 mg/kg/day) infusion. Tyrosine hydroxylase staining showed reduced tyrosine hydroxylase-positive cell in the substantia nigra (**D**). Note that TUNEL-positive neurons display brown color and were denoted by arrows (**E**) and caspase-3-positive neurons display brown color and were denoted by arrows (**F**). Scale bar, 50 μm.

**Figure 6:**
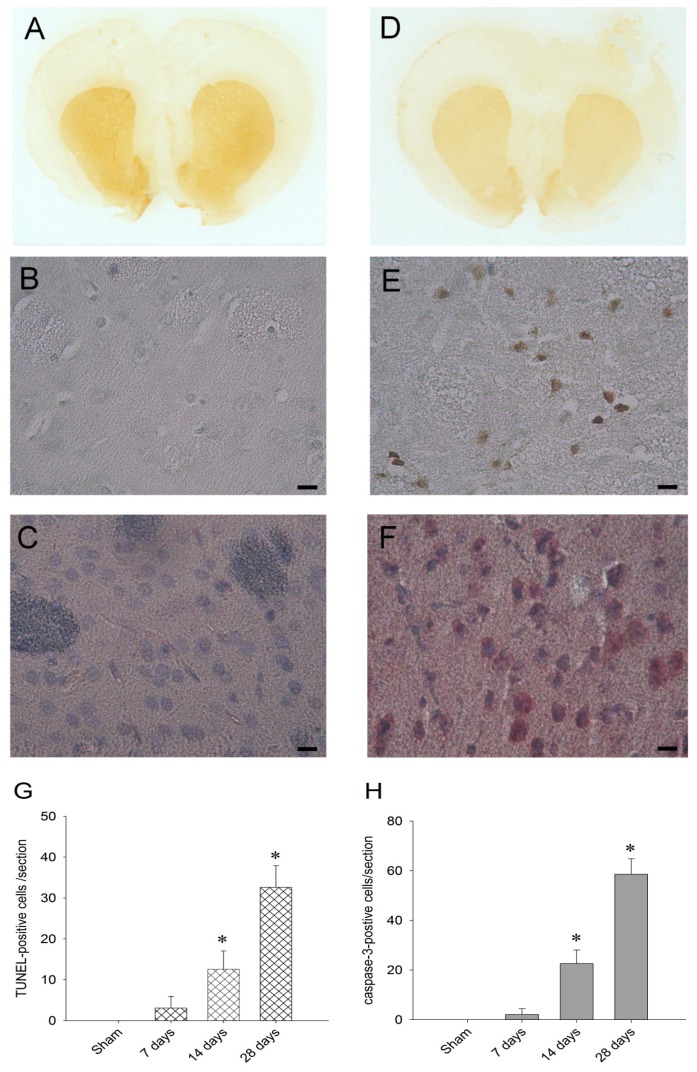
Tyrosine hydroxylase (**A**, **D**), terminal deoxynucleotidyl transferase-mediated dUTP-biotin nick end labeling (TUNEL) (**B**, **E**), and caspase-3 (**C**, **F**)-staining of the striatum in sham controls or 28 days of rotenone (3 mg/kg/day) infusion. Tyrosine hydroxylase staining showed reduced tyrosine hydroxylase-positive cell in the striatum (**D**). Note that TUNEL-positive neurons display brown color (**E**) and caspase-3-positive neurons display red color (**F**). Scale bar, 50 μm. Under quantitative assessment of neuronal cell death, TUNEL-positive cells (**G**) and caspase-3-postive cells (**H**) were counted. Values in (**G**, **H**) are mean ± SEM from 4 animals per experimental group. * *p* < 0.05 *versus* sham-control group.

After 28 days of rotenone (3 mg/kg/day) exposure, imunohistochemical staining for tyrosine hydroxylase showed reduced tyrosine hydroxylase-positive cells in the substantia nigra (Figure 5D) and striatum (Figure 6D), indicating that chronic rotenone intoxication resulted in nigrostriatal dopaminergic neuronal cell degeneration. However, the tyrosine hydroxylase staining in the stratum were relatively normal in sham-control animals (Figures 5A,6A). Moreover, TUNEL and caspase-3 staining were employed to confirm the neuronal nature of the apoptotic cells in the substantia nigra and striatum after chronic rotenone intoxication. TUNEL-positive cells (Figures 5E,6E) and caspase-3-postive cells (Figures 5F,6F) appeared on day 28 in the substantia nigra and striatum after systemic infusion of rotenone. On the other hand, TUNEL-positive and caspase-3-postive cells were essentially absent in the substantia nigra (Figure 5B,C) and striatum (Figure 6B,C) in the sham control animals. For quantitative assessment of neuronal cell death, striatal tissues were collected 7, 14, or 28 days after rotenone intoxication, and TUNEL-positive cells and caspase-3-postive cells were counted. Significant amounts of TUNEL-positive cells (Figure 6G) and caspase-3-postive cells (Figure 6H) appeared on day 14 and 28 in the striatum after systemic infusion of rotenone. The apoptotic neuronal cells were identified by morphology and size.

### 2.6. Discussion

The present study took advantage of an animal model of chronic systemic rotenone intoxication that closely resembles PD. Based on this animal model, the present study revealed that the repertoire of cellular events after inhibition of mitochondrial respiratory chain enzyme Complex I activity with chronic systemic rotenone infusion caused degeneration of the striatal neurons. Chronic rotenone intoxication entails inhibition of Complex I activity, followed by an excessive production of reactive oxygen species (ROS) and nitric oxide (NO) that induces mitochondrial dysfunction and ultrastructural damage, resulting in translocation of Bim and Bax from cytosol to mitochondria, release of mitochondrial cytochrome c to the cytosol that activates the caspase-3 cascade, leading to apoptotic cell death in the striatum.

Several biochemical abnormalities that were thought to be relevant to the pathogenesis were found in the brain of patients with PD [25]. Emerging evidence has suggested that mitochondrial dysfunction, increased oxidative stress, excitotoxicity, inflammatory changes and dysfunction of the ubiquitin-proteasome system may be involved in alpha-synuclein aggregation, Lewy body formation and neurodegeneration [21,25]. Mitochondrial dysfunction, particularly selective loss of Complex I activity and oxidative metabolism are critical components of most current theories of nigrostriatal degeneration in PD [5–7,25]. 1-methyl-4-phenyl-1,2,3,6-tetrahydropyridine (MPTP), a potent neurotoxin inadvertently used by heroin addicts results in inhibition of NADH-ubiquinone reductase activity which causes energy failure and neuron death in the substantia nigra and results in clinical symptoms of idiopathic PD suggested that mitochondrial dysfunction is responsible for the death of these dopaminergic neurons [26,27]. Since Complex I dysfunction plays a crucial role in the pathogenesis of PD, in the present study, we selected a specific mitochondrial Complex I inhibitor, rotenone, to induce dopaminergic cell death in the striatum and produce parkinsonian-like symptoms in the rats.

Rotenone is capable of destroying dopaminergic neurons. Findings from chronic, intravenous, or subcutaneous infusion of rotenone were reported to reproduce the major pathological and behavioral hallmarks of Parkinson’s disease, including selective degeneration of substantia nigra dopaminergic neurons, appearance of cytoplasmic inclusions reminiscent of Lewy bodies in degenerating neurons, and motor and postural deficits [17,19,20,28]. It has been reported that the neurodegeneration caused by continuous rotenone infusion is not restricted to substantia nigra dopaminergic neurons [18,22]. A high interindividual variability in the effectiveness of rotenone exposure to cause striatal degeneration in rats has also been suggested [22]. In a rotenone-treated animal model, neuron degeneration in the striatum and prefrontal cortex has been found [18]. In our present study, only those rats with evident motor symptoms were chosen to do the histology and biochemistry studies. Based on the immunohistochemical staining, we noted that a loss of dopaminergic neuronal cells both in the substantia nigra and striatum and some neurons exhibited TUNEL and caspase-3 positive staining. Therefore, we suggested that degeneration of nigrostriatal dopaminergic neuronal cells were significantly evident not only in the substantia nigra, but also in the striatum under chronic systemic rotenone exposure.

From its role as the cellular powerhouse, the mitochondrion is emerging as a key participant in cell death because of its association with an ever-growing list of apoptosis-related proteins [29,30]. Complex I is markedly more susceptible to oxidative stress and glutathionylation than other respiratory chain complexes [31]. As a major source of superoxide, it is a candidate for increasing mitochondrial reactive oxygen species (ROS) production and redox signaling [31,32]. There are also suggestions [33] that Complex I is involved in NO physiology, induction of the mitochondrial permeability transition, and regulation of apoptosis. In the present study, chronic systemic treatment of rats with rotenone, a well-established mitochondrial Complex I inhibitor, induces many key features of PD. These findings support the hypothesis of mitochondrial Complex I inhibition in the pathogenesis of PD. Furthermore, our study also showed the levels of oxidized proteins and nitrotyrosine were increased in the striatum following chronic systemic rotenone intoxication. Thus, inhibited activity of Complex I may increase production of ROS and peroxynitrite that can damage all cell structures, including lipids, proteins, DNA and mitochondrial membrane structure, leading to the striatal neuronal apoptosis [32–34].

Our electron microscopic examination of mitochondrial ultrastructure in the striatal neurons, which showed swelling and disruption of mitochondrial membrane that correlated temporally with chronic inhibition of mitochondrial respiratory chain enzyme Complex I function, offers further mechanistic insights on PD. Thus, both functional impairment and ultrastructural damage of mitochondria and increase of oxidative and nitrosative stress in the striatum may be a key to the pathogenesis of striatal neuronal cell death in this animal model of chronic rotenone intoxication. As inhibition of mitochondrial respiratory chain results in excess free radical production, and free radicals themselves are direct inhibitors of the mitochondrial respiratory chain, this can result in a vicious cycle that leads to oxidative cell damage [32,34].

One of the decisive steps of the apoptotic cascade is permeabilization of the outer mitochondrial membrane [35], which leads to the release of cytochrome c from the intermediate space, followed by the activation of caspase-dependent cascade of apoptotic signaling. Excessive NO and ROS affects oxidative phosphorylation by inhibiting the mitochondrial respiratory enzymes, and the resultant mitochondrial dysfunction induces apoptosis [32,34,36]. Complementary results from our biochemical analyses indicated that chronic systemic rotenone intoxication involved in the cytochrome c/caspase-3 apoptotic signaling cascade in the striatum.

It is generally contended that the anti-apoptotic members of Bcl-2 family work to prevent cytochrome c release by stabilizing the mitochondrial membrane barrier function and the pro-apoptotic members tend to induce cytochrome c release by permeabilizing the mitochondrial membrane [35]. Translocation of Bax from the cytosol to mitochondria is induced during apoptosis [35,37]. The evidence of Bcl-2 family involvement in Parkinson-linked genes and toxins induced striatal neuronal cell death has been demonstrated in recent studies, and both pro-apoptotic and anti-apoptotic Bcl-2 family proteins were found to be activated [38–45]. Oxidative stress or mitochondrial dysfunction induced by the neurotoxins MPTP, paraquat, maneb, and rotenone may cause cell death through members of the Bcl-2 family [39,41,44]. Sufficient activation of Bax facilitates mitochondrial outer-membrane permeabilization, which releases death-inducing factors that cause apoptotic and nonapoptotic programmed cell death [44]. In an experimental model of epileptic seizures, the proapoptotic factor Bim was upregulated by seizure activity that triggers Bax activation and translocation in the hippocampus [46]. In the present study, we observed the progressive translocations of cytosolic Bim and Bax to the mitochondria, alongside an increase in cytosolic presence of cytochrome c, suggesting that the Bim and Bax pathway had a key role in rotenone-induced apoptotic cell death via cytochrome c/caspase-3 signaling cascade in the striatum.

Based on histopathological analysis, we noted reduced tyrosine hydroxylase-positive cell in the striatum, which indicated that chronic rotenone intoxication resulted in nigrostriatal dopaminergic neuronal cell degeneration. Also, apoptotic cell death was detected in the striatal neurons after 28 days of systemic infusion of rotenone. It follows that prolonged exposure of neurons to rotenone toxicity may cause mitochondrial functional impairment and damages and increase of oxidative and nitrosative stress, leading eventually to apoptotic neuronal cell death in vulnerable regions of the striatum.

All experimental procedures were carried out in compliance with the guidelines for the care and use of experimental animals endorsed by our institutional animal care committee. All efforts were made to reduce the number of animals used and to minimize animal suffering during the experiment.

## 3. Materials and Methods

### 3.1. Animals

Experiments were carried out in specific pathogen-free adult male Lewis rats (300–350 g) that were obtained from the Experimental Animal Center of the National Science Council, Taiwan. They were housed in an animal room under temperature control (24–25 °C) and 12-h light-dark (08:00–20:00) cycle. Standard laboratory rat chow and tap water were available *ad libitum*.

### 3.2. Experimental Model of Parkinson’s Disease

An experimental animal model of PD that was induced by subcutaneously administration of a specific mitochondrial Complex I inhibitor rotenone was used [17]. This model entails a 2 mL of mini-osmotic pump (2ML4, ALZET, Cupertino, CA, U.S.A.) that was filled with rotenone (3 mg/kg/day) dissolved in dimethylsulfoxide (DMSO) and polyethylene glycol (PEG), which allows to slowly deliver rotenone for 28 days and generate chronic intoxication. The mini-osmotic pumps were placed in sterile 0.9% saline at 37 °C for at least 4 h before use. Osmotic mini pumps were subcutaneously implanted under the skin on the back after ketamine (75 mg/kg) and rompum (10 mg/kg) were given intramuscularly to induce anesthesia, and the rest of the body was placed on a heating pad to maintain body temperature at 37 °C. The wound was then closed in layers, and sodium penicillin (10,000 IU; YF Chemical Corporation, Taipei, Taiwan) was given intramuscularly to prevent postoperative infection. Animals were returned to the animal room for recovery in individual cages. Rats that implanted mini pumps with 2 mL of DMSO and PEG (*v*/*v* = 1:1) subcutaneously served as sham-controls. Rats were monitored for behavior, weight and overall health every day.

### 3.3. Collection of Tissue Samples from the Striatum

At pre-determined time-intervals (7, 14 or 28 days) after systemic infusion of rotenone by mini-osmotic pumps, rats were anesthetized with overdose pentobarbital (100 mg/kg, i.p.) and were perfused intracardially with 50 mL of warm (37 °C) saline that contains heparin (100 U/mL). The brain was rapidly removed under visual inspection and placed on a piece of gauze moistened with ice-cold 0.9% saline and the striata was routinely collected. The concentration of total proteins extracted from tissue samples was determined by the BCA Protein Assay (Pierce, Rockford, IL, USA). In selected experiments, proteins from the mitochondrial or cytosolic fraction of the striatal samples were extracted by a commercial kit (Active Motif, Carlsbad, CA, USA).

### 3.4. Western Blot Analysis

Western blot analysis for nitrotyrosine, an experimental index for peroxynitrite (Radi *et al.*, 2001), Bim, Bid, Bax, cytochrome c, nitrotyrosine or β-actin was carried out on proteins extracted from mitochondrial or cytosolic fractions of striatal samples [47–49]. The purity of the mitochondrial fraction was verified by the selective expression of the mitochondrial inner membrane specific protein, cytochrome c oxidase subunit IV (COX IV). Protein concentration was determined by the BCA Protein Assay (Pierce). The primary antisera used included rabbit polyclonal antiserum against Bim, Bid, Bax and COX IV (Cell Signaling, Danvers, MA, USA), mouse monoclonal antiserum against cytochrome c (Santa Cruz Biotechnology, Santa Cruz, CA, USA) and nitrotyrosine (Upstate, Lake Placid, NY, USA). The secondary antisera used included horseradish peroxidase-conjugated sheep anti-mouse IgG (Amersham Biosciences, Little Chalfont, UK) for cytochrome c, nitrotyrosine and β-actin, or donkey anti-rabbit IgG (Amersham Biosciences) for Bim, Bid, Bax, and COX IV. Specific antibody-antigen complex was detected by an enhanced chemiluminescence western blot detection system (NEN, Boston, MA, USA). The amount of protein was quantified by the ImageMaster software (Amersham Pharmacia Biotech, Piscataway, NJ, USA), and was expressed as the ratio relative to β-actin protein (for analysis of total protein or proteins in cytosolic fraction) or COX IV (for analysis of proteins in mitochondrial fraction).

### 3.5. Detection of Oxidized Proteins

Oxidized protein was detected by using a protein oxidation detection kit (OxyBlot, Chemicon, Temecula, CA, USA) [50]. This kit provides reagents for sensitive immunodetection of carbonyl group, which is a hallmark of the oxidation status of proteins [51]. Total proteins extracted from the striatal area at various time points after exposure to rotenone were reacted with 2,4-dinitrophenylhydrazine and derivatized to 2,4-dinitrophenylhydrazone (DNP-hydrazone) [52]. The DNP-derivatized protein samples were separated on a 15% SDS-polyacrylamide gel followed by western blotting. The blot was incubated with a primary antibody with rabbit anti-DNP antibody and followed by incubation with a horseradish peroxidase-conjugated goat anti-rabbit IgG secondary antibody according to manufacturers’ instructions.

### 3.6. Electron Microscopy

After 28 days of rotenone intoxication, striatum was removed and processed for electron microscopy. Tissue samples were diced and submerged in 4% glutaraldehyde (0.1 M sodium cacodylate buffer, pH 7.2). Tissues were postfixed with osmium, and en bloc stained with uranyl acetate. After dehydration, each specimen was embedded by infiltration in Spurr’s medium. Following trimming of the tissue blocks, sections were cut to a thickness of 90 nm, post-stained with uranyl acetate and lead citrate, and viewed on 300 mesh-coated grids using a JEOL JEM-1230 (Tokyo, Japan) electron microscope [50,53,54].

### 3.7. Immunohistochemical Staining

To evaluate the dopaminergic neuronal loss in the striatum and substantia nigra following chronic rotenone intoxication, imunohistochemical staining for tyrosine hydroxylase was performed. The rats were overdosed with pentobarbital (100 mg/kg, i.p.) after 28 days of the chronic rotenone intoxication for immunostaining. Intra-cardiac perfusion was performed with 50 mL of warm (37 °C) saline that contains heparin (100 U/mL), followed by 400 mL of 4% paraformaldehyde in 0.1 M PBS for tissue fixation. The forebrains were carefully dissected, and a segment containing the striatum were blocked and fixed for additional 2 h in the same fixatives, and transferred to a solution containing 30% sucrose in 0.1 M PBS. The brain sections were then embedded in tissue freezing medium (Sakura Finetek, Torrance, CA, USA), serially sectioned in the coronal plane throughout the rostral-caudal extent at 7-μm interval on a cryostat, and mounted on Superfrost/plus slides (Fisher Scientific, Pittsburgh, PA, USA). Before incubation with primary antibodies, the sections were permeabilized with 0.3% Triton X-100 and 10% horse serum in 0.01 M PBS for 20 min. A monoclonal mouse antibody against tyrosine hydroxylase (Chemicon, Temecula, CA, USA) was applied to the sections overnight at 4 °C. The following day the brain sections were incubated with a secondary biotinylated goat anti-mouse immunoglobulin G (IgG) (Vector Laboratories, Burlingame, CA, USA).

As histochemical markers for apoptotic cell death, animals were processed for terminal deoxynucleotidyl transferase-mediated dUTP-biotin nick end labeling (TUNEL) and caspase-3 staining to examine the apoptotic cell death in the striatum and substantia nigra following experimental animal model of Parkinson’s disease. Paraffin-embedded sections of the striatum were also processed for TUNEL and staining using an *in situ* apoptosis detection kit (ApopTag, Intergen Company, Purchase, NY, USA) and a rabbit polyclonal antiserum against activated caspase-3 (Cell Signaling) after 28 days of rotenone intoxication. The tyrosine hydroxylase-positive, TUNEL-positive or caspase-3-positive cells on each section were viewed under an Olympus AX70 microscope [48,54]. TUNEL-positive or caspase-3-positive cells were counted in a double-blind manner in the middle region of the striatum.

### 3.8. Statistical Analysis

All values are expressed as mean ± SEM. One-way analysis of variance (ANOVA) was used, as appropriate, to assess group means, followed by the Scheffé multiple-range test for post hoc assessment of individual means. *p* < 0.05 was taken to indicate statistical significance.

## 4. Conclusions

The present study demonstrated that chronic inhibition of Complex I activity by systemic infusion of rotenone may cause an excessive production of ROS and NO that induces mitochondrial dysfunction and ultrastructural damage, resulting in translocation of Bim and Bax from cytosol to mitochondria that triggers mitochondrial cytochrome c release to the cytosol and initiates caspase-3-dependent apoptotic cell death in the striatum. Therefore, to understand the mechanism of neuronal death in the vulnerable neurons during the process of PD, this study might offer novel prospects for therapy based on targeted neuroprotection.
